# Male-biased gastrointestinal parasitism in a nearly monomorphic mountain ungulate

**DOI:** 10.1186/s13071-015-0774-9

**Published:** 2015-03-18

**Authors:** Jordi Martínez-Guijosa, Carlos Martínez-Carrasco, Jorge Ramón López-Olvera, Xavier Fernández-Aguilar, Andreu Colom-Cadena, Oscar Cabezón, Gregorio Mentaberre, David Ferrer, Roser Velarde, Diana Gassó, Mathieu Garel, Luca Rossi, Santiago Lavín, Emmanuel Serrano

**Affiliations:** Departamento de Sanidad Animal, Facultad de Veterinaria, Universidad de Murcia, Murcia, Spain; Servei d’Ecopatologia de Fauna Salvatge (SEFaS), Wildlife Health Service - Departament de Medicina i Cirugia Animal, Universitat Autònoma de Barcelona, Bellaterra, Spain; Departament de Sanitat i d’Anatomia Animals, Bellaterra, Universitat Autònoma de Barcelona (UAB), Bellaterra, Spain; Centre National d’Études et de Recherche Appliquée Faune de Montagne, Office National de la Chasse et de la Faune Sauvage (ONCFS), Juvignac, France; Dipartimento de Produzioni Animali, Epidemiologia ed Ecologia, Universtità di Torino, Turin, Italy; Departamento de Biologia & CESAM, Universidade de Aveiro, Aveiro, Portugal

**Keywords:** Co-occurrence, Multiparasitism, Null models, Parasite communities, Sexual size dimorphism, *Rupicapra pyrenaica*

## Abstract

**Background:**

Pyrenean chamois (*Rupicapra pyrenaica pyrenaica*) is a nearly monomorphic mountain ungulate with an unbiased sex-specific overwinter adult survival. Few differences in gastrointestinal parasitism have been reported by coprology as yet. This study aims to assess diversity, prevalence, intensity of infection and aggregation of gastrointestinal nematodes in male and female adult chamois. We expect no differences in the parasite infection rates between sexes.

**Findings:**

Gastrointestinal tracts of 28 harvested Pyrenean chamois in the Catalan Pyrenees (autumn 2012 and 2013) were necropsied and sexual differences in the diversity and structure of parasite community, prevalence, intensity of infection, and richness were investigated. We found 25 helminth species belonging to 13 different genera.

**Conclusions:**

Contrary to our expectations, male chamois showed different parasite communities, higher prevalence, intensity of infection and richness than females. Such sexual differences were clear irrespective of age of individuals. Hence, male chamois must cope with a more diverse and abundant parasite community than females, without apparent biological cost. Further research will be required to confirm this hypothesis.

## Findings

Sex-biased parasitism has been linked to a higher susceptibility of helminth infection in males of a broad range of mammal species [1]. Once infected, this greater male-biased susceptibility is primarily driven by the effects of immunosuppressive hormones (i.e., testosterone [2,3]) and differences in energy and nutrient requirements for parasite defence [4]. Hence, under stressful environmental conditions (e.g., food shortage) resilience of male hosts against parasitism may be lower than for females.

In temperate ecosystems, higher energetic demands occur in winter when a period of reduced availability coincides with increased thermoregulatory demands [5]. The energetic requirements will be even higher if the rut coincides with the decrease in food availability as in most ungulate species (e.g., Caprinae) inhabiting Alpine ecosystems [6]. On the other hand, rut-induced hypophagia, the reduction in time spent foraging during the mating season, of males in these mammals [7], may increase susceptibility to parasite infection due to the high testosterone concentration and the reduction in food intake.

The Pyrenean chamois (*Rupicapra pyrenaica pyrenaica*) is a nearly monomorphic mountain ungulate that experiences much of the previously mentioned characteristics linked to male-biased parasitism. In this caprinae, rut begins at the end of October and lasts until December [8] coinciding with a period of diet impoverishment [9]. In addition, seasonal changes in androgen metabolites match the sexual cycle of this mammal, and an increase in lung nematode load in males [10]. However, this male-biased parasitism has not been fully confirmed for gastrointestinal helminths [10] and the increments in lung nematode loads are not widespread depending on the mating tactic, i.e., only territorial males but not all had greater lung nematode loads [11]. On the other hand, this slightly sex-biased susceptibility to parasite infection has been assessed by indirect counts (i.e., coprology) and to date no research has been conducted to study whether this male-biased parasitism is due to the higher reproduction rates of a few parasite species or to a more abundant and diverse parasite community. Host are considered as complex ecosystems composed of parasites [12], hence male-biased parasitism should be explored considering as much as the endoparasites community as possible. Accordingly, in this work we (i) identify the gastrointestinal helminth species affecting male and female Pyrenean chamois during the rut, and (ii) examine whether or not sexual differences, in terms of prevalence, intensity of infection, diversity and community structure of gastrointestinal helminths, exists in different digestive regions (i.e., abomasum, small intestine and large intestine) of chamois’ gastrointestinal tract.

## Material and methods

### Chamois sampling

Gastrointestinal tracts (n = 28) were obtained by necropsy of 17 female (3–16 years old) and 11 adult male (2–12 years old) Pyrenean chamois from the Freser-Setcases National Game Reserve, Catalan Pyrenees, Spain (4°21′N, 2°09′E). Animals were hunter-harvested during October-December 2012 and 2013 coinciding with the rut period. Age determination was based on horn annuli counts. Once the gastrointestinal tract was removed, we tied the abomasum, and small and large intestine ends. The gastrointestinal tract was then stored in labelled plastic bags and transported in a cold box at 4°C to our facilities. In the laboratory, the material was stored at -20°C until parasitological examination.

### Parasitological data

Once gastrointestinal tracts were defrosted, the abomasum, and small and large intestine were longitudinally opened (n = 84), the mucosa scrapped and the content washed and filtered through three sieves of 6.3, 3.2 and 0.3 mm, respectively. The content was diluted in 1000 ml of tap water in a sedimentation cup and three aliquots of 100 ml (10%) each were examined to collect parasites. Male nematodes were cleared in lactophenol and Cestodes stained in Semichon’s carmine and later identified.

### Statistical analyses

For nematodes, prevalence and intensity of infection (number of parasite individuals/number of infected hosts) were calculated whereas for cestodes, only prevalence was estimated.

We used null models to assess parasite diversity and to explore whether gastrointestinal parasite species in female and male chamois were occurring in structured communities. For parasite diversity we used both the species richness (number of gastrointestinal parasite species per individual chamois) and the PIE Hurlbert’s index (i.e., the probability that two randomly sampled parasites from the host population belong to different species). To assess co-occurrence among parasite species we used the C-score index. Low C-score values mean that species frequently occur together and hence a C-score smaller than expected by chance (O<E) indicates positive co-occurrence, e.g., species in that community will tend to be aggregated [13]. The fixed-equiprobable (F-E) algorithm was used, and a standardised effect size (SES) for each matrix (i.e., number of standard deviations that the C-score is above or below the mean index of the simulated communities) calculated. Expected C-scores were estimated for 5000 null matrices by Monte Carlo procedures using EcoSim 7.72 [14].

Sex differences in mean prevalence of specific gastrointestinal helminth infections (as response variable) were assessed using linear models (LM). In these models, sex, digestive region (i.e., abomasum, small intestine and large intestine) and their interaction were considered as explanatory variables. On the other hand, male-biased helminth richness or intensity of infection was also evaluated using LM including the age (in years), sex, digestive portion and their interaction as explanatory variables. Host individual was initially included as random factor (intercept) in a linear mixed model, but later excluded since it was not statistically significant. Model selection was assessed by the Akaike Information Criterion [15]. Richness and mean intensity of infection was log-transformed to minimise the residual pattern. Prior to model interpretation, model requirements were evaluated according to Zuur et al. 2013. Analyses were performed in R, version 3. 1. 2 [16].

## Results and discussion

Prevalence of gastrointestinal helminth infection was 96.5% and a single female was infection-free. The number of parasites per individual ranged from 0 to 6500 helminths. Twenty-five helminth species were identified, 9 species in both the abomasum and small intestine whereas 7 were identified in the large intestine (Table 1).

**Table 1:** **Prevalence, mean intensity and range (min-max) of gastrointestinal helminth infections in Pyrenean chamois (17 ♀ and 11 ♂) hunter-harvested in the Freser-Setcases National Game Reserve, Catalonia, Spain**

	**Prevalence**		**Mean intensity**		**Range**	
**Abomasum**	**♀**	**♂**	**♀**	**♂**	**♀**	**♂**
*Haemonchus contortus*	43.7	81.8	55.1	59.5	0–147	0–242
*Trichostrongylus axei*	50.0	100	178.2	246.7	0–971	18–998
*Teladorsagia circumcincta*	62.5	90.9	194.1	414.3	0–695	0–1158
*Teladorsagia trifurcata*	43.7	90.9	37.2	95.6	0–137	0–378
*Ostertagia leptospicularis*	18.7	54.5	10.7	285.9	0–17	0–1344
*Ostertagia ostertagi*	6.2	54.5	8.4	194.5	0–8	0–836
*Ostertagia lyrata*	0	9.1	0	31.2	0–0	0–31
*Marshallagia marshalli*	56.2	90.9	115.2	334.1	0–374	0–974
*Marshallagia occidentalis*	37.5	72.7	19.4	111.7	0–37	0–413
*Total*	87.5	100	371.9	1408.9	0–1292	37–3678
**Small intestine**						
*Trichostrongylus capricola*	11.7	18.2	8.6	25.3	0–17	0–28
*Trichostrongylus colubriformis*	11.7	27.3	32.2	24.8	0–53	0–38
*Trichostrongylus vitrinus*	35.3	27.3	18.6	15.8	0–40	0–22
*Nematodirus oiratianus*	58.8	100	280.9	1137.9	0–1254	75–3059
*Nematodirus filicolis*	41.2	100	102.1	206.1	0–366	5–517
*Nematodirus abnormalis*	0	18.2	0	86.6	0–0	0–149
*Capilaria bovis*	29.4	18.2	4.6	6.7	0–7	0–10
*Cooperia oncophora*	0	9.1	0	47.8	0–0	0–48
Cestodes	11.7	45.4	-	-	-	-
*Total*	76.5	100	191.7	1381.1	0–1120	80–3382
**Large intestine**						
*Oesophagostomum venulosum*	64.7	81.8	2.4	4.8	0–6	0–14
*Trichuris globulosa*	11.7	9.1	1	1	0–1	0–1
*Trichuris ovis*	5.9	36.4	3	1.5	0–3	0–2
*Trichuris discolor*	5.9	9.1	2	1	0–2	0–1
*Trichuris skrjabini*	5.9	0	1	0	0–1	0–0
*Skrjabinema ovis*	0	9.1	0	4	0–0	0–4
*Chabertia ovina*	0	9.1	0	1	0–0	0–1
*Total*	64.7	81.8	3.1	6.2	0–8	0–16
**Total**	93.7	100	489.8	2795.1	0–2209	120–6500

Only 3 of the 7 cestodes found were classified as *Moniezia* sp.

In both sexes, the highest probability of an inter-specific encounter (PIE) occurred in the abomasum and the lowest in the small intestine (Table 2). The observed C-score (Table 2) was smaller than that expected by chance (O>E) in every digestive portion, indicating that parasites were organised in communities.

**Table 2:** **Parasite community diversity and aggregation analysis of Pyrenean chamois (17 ♀ and 11 ♂) from Freser-Setcases National Game Reserve, Catalonia, Spain**

**Digestive portion**	**Richness**		**PIE**		**C-score**							
	♂	♀	♂	♀	♀				♂			
					**O**	**E**	**P**	**SES**	**O**	**E**	**P**	**SES**
Abomasum	9	8	0.83	0.74	6.14	13.02	<0,001	-4.08	1.11	3.68	0.000	-4.86
Small intestine	9	7	0.40	0.30	8.29	9.89	0.006	-2.77	1.97	3.04	0.026	-2.15
Large intestine	6	5	0.40	0.40	0.40	2.94	0.004	-2.17	0.93	2.12	0.05	-1.74

Average richness, mean probability of an interspecific encounter (PIE, Hurlbert’s 1971), and observed (O) and expected by chance (E) values of the C-score for presence/absence matrices. The P indicates the p-value (O<E). Negative values of the standardised effect size (SES) indicate that O < E.

**Figure 1:**
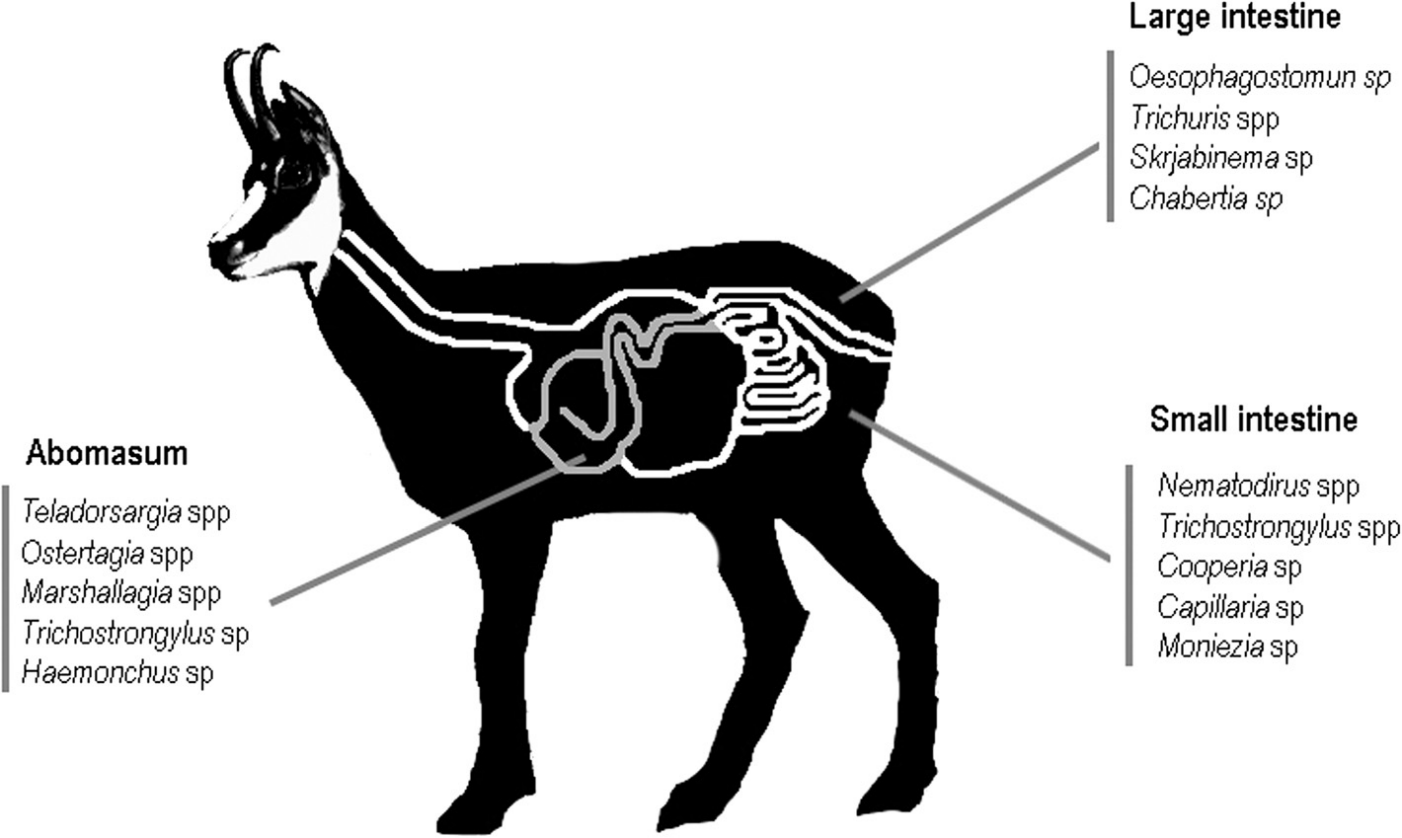
**Gastrointestinal helminth community of Pyrenean chamois, a monomorphic mountain ungulate.** Helminth genus have been ordered according to decreasing order of mean intensity of infection. Omasum, abomasum and first portion of the small intestine (grey solid line) are placed on the right size.

The additive effects of sex and digestive region were sufficient to explain the observed variability in prevalence of gastrointestinal helminth infection (F_3, 46_= 7.6, p < 0.01, R^2^= 33.3%). In fact, a male-biased mean prevalence (46% in males vs 24% in females) was clear in our sample of chamois. Regarding the intensity of infection, 64% of the observed variability was due to the effects of age, digestive portion and the sex of chamois (F_4, 75_ = 33.39, p < 0.01). In both sexes, intensity of infection decreased with age (β= -0.06, SE = 0.03, t= -2.2, p = 0.01), being higher in males (β= 1.52, SE = 0.28, t= 5.3, p = 0.02) than in females (133.8 helminths /individual host in males vs 42 helminths/individual host in females). Interestingly, in both sexes the greatest intensity of helminth infections were found in the abomasum (133 helminths/host individual), followed by the small intestine (111 helminths/host individual) and lastly the large intestine (1.6 helminths/host individual). A picture summarising the parasite distribution in an adult chamois can be seen in Figure 1. Along the same lines, 55.1% of the observed helminth richness variability was also due to the effects of age, sex and the digestive portion (F_4, 75_ = 23.12, p < 0.01). Richness also decreased with age (β= -0.01, SE = 0.02, t = -2.71, p < 0.01), males hosted more parasite species than females (β= 0.12, SE = 0.02, t = 4.6, p < 0.01), and the abomasum was richer in species than the small or the large intestine. In addition, the PIE tended to be sex-biased, being higher in males in the abomasum and small intestine (Table 2). These considerable variations among digestive segments are common and likely because of the greater nutrient availability in the first two portions; however, no information exists to support this hypothesis.

These sexual differences were mainly due to the higher infection rates of *Ostertagia* spp*.*, *Teladorsagia* spp., *Nematodirus* spp*.,* and *Marshallagia* spp*.* in males (Table 1)*.* Actually, one helminth species (*Trichuris skrjabini)* was not found in males whereas five helminth species (*Ostertagia lyrata, Skrjabinema ovis, Chabertia ovina, Cooperia onchophora* and *Nematodirus abnormalis*) where not found in female chamois.

We found the first record of *C. oncophora*, *Ostertagia leptospicularis*, *O. lyrata* and *Trichuris discolor* in the Southern chamois and the first report of *O. ostertagi* in chamois from the Iberian Peninsula. Interestingly, we found several parasite species common to livestock (e.g., *N. filicolis*, *C. oncophora*, *H. contortus*, *C. ovina*, and *Teladorsagia* spp., [17]), highlighting the risk of cross-infections with sheep sharing alpine pastures.

Our co-occurrence analysis indicates that gastrointestinal parasites in each digestive part are organised in structured assemblages (i.e., not a random combination of helminth species) in both female and male chamois. There is no consensus however regarding the meaning of interspecific interactions among parasites in these structured communities, but the most plausible hypothesis is that the structuring process is based on competitive interactions, i.e., the exclusion of one species by another, between nematodes [18].

Furthermore and contrary to our expectations, the male-biased gastrointestinal helminth prevalence, intensity of infection and richness was manifest at any age. Few differences in the behaviour of males can partially explain the differences in the parasitization rates. In the summer, male chamois have larger home ranges [19] and feed at greater intensities [20] than females coinciding with the peak of infective L3 larvae in the alpine meadows [21]. This behaviour not only allows males to achieve more accumulated body resources for the rut period [22], but may also influence the likelihood of acquiring new parasite species by accidental ingestion of infective larvae. Subsequently, the energetic demand of rut, the rut-induced hypophagia, and the increased concentration of androgens [23] may favour the establishment of the helminth infections. Interestingly, this male-biased parasitism is also detectable outside of the rut period [24] and hence this establishment of new infections during the rut period would result in a greater bioaccumulation of parasites in male chamois.

## Conclusions

This study confirms male-biased parasitism in the Pyrenean chamois, despite the low sexual dimorphism in this mammal. However, and in contrast to other polygynous ungulates, survival patterns of female and male Pyrenean chamois are similar [25,26]. Hence, this sex-biased parasitism may not implicate a greater biological cost for males with respect to females. Further research will be oriented to test this supposition.
